# The benefits of in situ reporting: Experimental evidence from in-the-moment surveys of public transit riders

**DOI:** 10.3758/s13428-026-03022-z

**Published:** 2026-05-26

**Authors:** Christopher Antoun, Vanessa Frías-Martínez, Anthony Garove, Naman Awasthi, Saad Mohammad Abrar

**Affiliations:** 1 College of Information, University of Maryland, 4130 Campus Drive, College Park, MD 20742 USA; 2 Joint Program in Survey Methodology, University of Maryland, College Park, MD USA; 3 Department of Computer Science, University of Maryland, College Park, MD USA

**Keywords:** In-the-moment surveys, Real-time data collection, *In situ* measurement, Survey Incentives, Smartphone surveys

## Abstract

In-the-moment surveys administered when respondents participate in a predetermined event of interest—such as visiting a particular location or completing a specific activity—allow researchers to measure behaviors and experiences as the event occurs, situating data collection within the relevant context and reducing reliance on retrospective self-reports. However, data quality can be compromised if individuals are unwilling to participate or provide low-quality responses. In particular, the timing of survey prompts and the structure of incentives may affect data quality. We evaluated these issues in an experimental study of 610 individuals who used a custom smartphone app to answer a survey about each of their public transit trips over 2 weeks. Participants were randomly assigned to receive either daily or per-survey incentives, with surveys administered either during trips (in situ) or immediately afterward (near-time). Both daily incentives and in situ prompts significantly increased participation. In situ prompts also reduced “speeding” and increased the variation in survey responses across trips, suggesting improved accuracy in capturing event-specific details. These findings demonstrate that prompt timing—specifically, in situ prompts as opposed to near-time prompts—plays a key role in enhancing data quality for in-the-moment surveys.

## Introduction

Social researchers are increasingly interested in survey methods that measure respondents’ behaviors and experiences in context as specific events occur, rather than relying on retrospective reports. The widespread use of smartphones has accelerated this trend, making it feasible to administer survey questions via specialized apps during or very soon after relevant events. However, there remain concerns about data quality, as individuals may be unwilling to participate or provide low-quality responses. Furthermore, even surveys completed shortly after an event may still be subject to memory and recall issues, which real-time data collection seeks to prevent.

Our study addresses these challenges by examining two key design decisions that could affect data quality: the structure of incentives and the timing of survey invitations. The first focuses on optimizing incentives to improve response rates. Incentives in surveys administered repeatedly (e.g., each time the respondent participates in an event of interest) are commonly provided to respondents based on the number of surveys they complete during the study (Degroote et al., 2020). We examine an alternative approach that incentivizes daily participation, aiming to encourage consistent engagement throughout the study period. The second design consideration involves the strategic timing of survey invitations (i.e., prompts). Conventionally, when prompts are associated with an event of interest, they are sent immediately (or shortly) after the event (Bolger & Laurenceau, 2013); our study evaluates the effectiveness of issuing prompts during the event itself.

We evaluated these design strategies in a study that invited participants to use a custom smartphone app to report on their experiences riding public transit over a period of 2 weeks. Using a 2 × 2 randomized factorial experiment, incentives were offered either per survey completed or per day of participation (both capped at the same maximum amount), and survey prompts were sent either during the transit ride (in situ) or immediately afterward (near-time).^1^ Our primary research question is how these strategies affect survey completion rates and response quality, with the goal of enhancing our understanding of how design factors impact data quality and improving the effectiveness of survey methods for assessing participants’ experiences as they occur.

### Background

Methods of momentary data capture—such as ecological momentary assessment (EMA), experience sampling method (ESM), and ambulatory assessment (AA)—allow researchers to prompt participants to report their current experiences, feelings, and behaviors within their natural environments (Bolger & Laurenceau, 2013; Csikszentmihalyi & Larson, 1987; Doherty et al., 2021; Eisele et al. 2022; Stone et al., 1998). In this article, we focus on a particular subset of these methods: surveys administered in connection with a predetermined event of interest (e.g., visiting a specific location, completing a specific activity). These surveys are referred to using different terms, such as event-based EMA if administered repeatedly (distinct from time-based EMA, Shiffman et al., 2008) or in-the-moment (ITM) surveys (Ochoa & Revilla, 2023); for consistency, we use the term ITM. This approach enables measurement to occur during or immediately following the target event, situating data collection in the context where the event unfolds, rather than in a different setting (e.g., at home or much later), as is common in conventional surveys. Accordingly, ITM surveys typically include questions that assess current, rather than past, behaviors or experiences. The surveys can vary both in how the target event is detected (e.g., through passive sensing, manual entry by participants) and in the timing of survey prompts relative to the event (e.g., during the event, immediately afterwards).

ITM surveys are now used to collect data about a broad range of events. For example, Kreuter et al. (2020) deployed surveys to job seekers immediately after they visited job centers, collecting details about their activities. Wray et al. (2019) triggered surveys to collect information about respondents’ drinking behavior while they were visiting popular bars. Sugie (2018) administered brief surveys to men recently released from prison, prompting them to report on their social interactions upon receiving calls or text messages from new phone numbers.

Despite these varied applications, there is a paucity of methodological research on how to optimally design ITM surveys. Specific design features—particularly the structure of incentives and the timing of survey prompts—may play key roles in enhancing data quality, yet their impacts have not been systematically examined and may vary across study contexts. In the following sections, we draw on a small but growing body of research to describe how these factors might influence participation rates and response quality.

### Incentive structure

For ITM surveys, as with cross-sectional surveys, research has shown that performance-based incentives can positively affect response rates (Ottenstein & Werner, 2022; Wrzus & Neubauer, 2023). Social exchange theory helps explain this phenomenon, suggesting that individuals are more inclined to participate when they perceive the benefits as outweighing the costs, with incentives enhancing the perceived benefits (Dillman et al., 2014; Singer & Ye, 2013).

However, the optimal structure for incentive payments for ITM surveys—which can differ from traditional surveys by being repeated over time, sometimes more than once per day—remains uncertain. A common approach involves offering incentives based on the number of surveys completed. This includes a reward per survey question (e.g., £0.10 per answered question, Haas et al., 2020) or for reaching a specified response rate (e.g., $15 for completing at least 75% of surveys, Sugie, 2018). Alternatively, incentives can be tied to the length of time in which an individual participated (e.g., £50 per day, Jäckle et al., 2019).

To our knowledge, these two approaches have not been directly compared in an experimental study. When multiple survey invitations can be sent each day, daily incentives would provide a higher payout per day than the per-survey incentives would provide for each survey. For example, in our 2-week study, participants could earn $4 per day of participation, compared with $2 per completed survey, with a total maximum incentive of $56 for both conditions. This structure means that the first survey of each day offers a greater reward for the daily incentive structure, possibly encouraging participants to engage consistently throughout the study, which is critical for researchers interested in measuring changes in outcomes over time. This logic aligns with Jäckle et al.'s (2019) finding that offering a daily incentive in a study where participants used an app to record their spending resulted in relatively consistent participation over 30 days.

Conversely, daily incentives limit additional earnings on a given day after completing the first survey, whereas per-survey incentives offer extra rewards for each additional survey completed that day. Thus, if participants are invited to participate in multiple surveys per day (e.g., if they take multiple trips), per-survey incentives might encourage them to complete more surveys, benefiting studies aiming to maximize the total number of surveys completed (rather than consistent participation over time). On this basis, we propose the following hypotheses:

- **H1:** Daily incentives will increase the number of days participants complete a survey.
- **H2:** Per-survey incentives will increase the total number of surveys completed.

With respect to response quality, previous studies have shown that incentives primarily serve as motivation for participation rather than improving the quality of data provided (Giese & König, 2024; Singer & Ye, 2013). In line with this research, we do not expect the incentive structure to have a noticeable effect on response quality.

### Survey timing

In addition to the incentive structure, this study investigates the timing of survey prompts for ITM surveys. Typically, participants are asked to complete surveys immediately after an event has occurred (Bolger & Laurenceau, 2013, pp. 11–26; Tate et al., 2024). In the context of transit surveys, passengers might be asked to complete a survey after their trip or intercepted as they disembark from a bus or train (Schaller, 2005). However, advancements in apps and sensor technology have made it easier to trigger surveys during events themselves, that is, in situ (Ebner-Priemer et al., 2013; Giurgiu et al., 2020; Keusch & Conrad, 2022; Reichert et al., 2020; Törnros et al., 2016). In the context of transit surveys, this is akin to conducting onboard surveys (Schaller, 2005). Despite this technological capability, to our knowledge no prior studies have experimentally compared these two prompt delivery strategies.

In determining the effectiveness of in situ prompts, a key consideration is how likely participants are to complete surveys that are triggered during an event. Research indicates that this likelihood is influenced by the nature of the activity in which participants are engaged. They are more likely to respond when engaging in activities with low cognitive demand and that are easily interruptible, such as walking or using social media, than when engaging in activities such as gaming and in-person social interactions (Ochoa & Revilla, 2023; Ochoa Gómez, 2022; Yan et al., 2021). For regular public transit passengers, riding a bus/train may be perceived as a routine activity that requires minimal cognitive effort and is conducive to interruptions. For example, one study reported that most seated public transit riders often engage in other tasks, such as using their phones (Liang & Hwang, 2016), suggesting that they can shift to activities such as completing app-based surveys without significant disruption.

However, the specific context of transit riders can influence their survey-taking experience. Cognitive demand may increase during peak commuting times (rush hour), such as during weekday morning and afternoon periods, when buses and trains tend to be more crowded, and seating is limited. In such situations, near-time prompts might be advantageous, as they allow participants to complete surveys at a more convenient time within a restricted window (no more than 30 min after the event). Considering these factors, we propose the following hypothesis:

- **H3**: Compared with near-time prompts, in situ prompts will increase the survey response rate. This effect may be moderated by the time of day, as in situ surveys might be more difficult to complete during peak hours (rush hour) than during off-peak times.

Another important consideration is whether asking questions during an event will improve response quality. When answering questions requires substantial effort, respondents may engage in satisficing, which involves taking cognitive shortcuts to produce an answer that is satisfactory rather than optimal (Krosnick, 1991). This can manifest in various response strategies, such as answering so quickly that it is unlikely that questions have been adequately processed (speeding) and selecting the same response option for all questions in a battery (straightlining) (Zhang & Conrad, 2014). Research suggests that respondents are less likely to satisfice when response tasks are less cognitively demanding, other things being equal (Galesic & Bosnjak, 2009). For ITM surveys, answering questions during an event might be easier because individuals can respond based on their immediate environment without needing to retrieve information from memory (Tourangeau et al., 2000; Willis et al., 1991).

Nonetheless, the specific context for respondents can again influence their experience. During peak public transit hours, crowded buses or trains with limited seating might create a distracting environment, making it more difficult to complete surveys during a trip than afterwards. In contrast, during nonpeak hours, when there are fewer passengers and more available seats, the environment may be less distracting. Thus, we propose our next hypothesis:

- **H4**: Compared with near-time prompts, in situ prompts will reduce satisficing behaviors. This effect may be moderated by the time of day, as in situ surveys might be more difficult to complete during peak hours (rush hour) than during off-peak times.

Beyond issues related to satisficing, our final consideration is whether asking questions during an event will improve the accuracy of self-reports. Research on the cognitive response process indicates that during an event, respondents have immediate access to their sensory experiences and granular details about the experience. However, as time passes, the accuracy of these memories diminishes, leading participants to generalize based on their typical experiences (Christensen et al., 2003; Schwarz, 2012; Schwarz et al., 2009; Wu et al., 2020). In our study, varying responses by the same respondent from trip to trip would suggest that the respondent is reporting unique details each time. Conversely, if a respondent provides the same answers repeatedly, it suggests that they are relying on generalizations that overlook trip-specific details. We label the extent to which a respondent's answers vary from trip to trip as *response differentiation*.

While our study sends near-time prompts directly after trips to mitigate recall issues, evidence indicates that even brief delays can cause memories to degrade for routine events (Means & Loftus, 1991). For example, in a time-use study, Al Baghal et al. (2014) found that respondents forgot specific details about their activities when reporting them the following day. Similarly, van Berkel et al. (2019) reported that the accuracy of self-reports about mobile phone usage, such as which applications were used, decreased after a delay of just 1–2 h. Hence, our final hypothesis is as follows:

- **H5**: Compared with near-time prompts, in situ prompts will lead to greater response differentiation across trips. 

While in situ prompts are expected to improve the accuracy of reports about respondents’ immediate experiences, they may miss details if conditions change after survey completion but before the event ends. For this reason, such prompts are best suited for measuring aspects that remain relatively stable throughout the event, rather than those that might change or depend on the event’s conclusion.

In summary, there is limited understanding of how different incentive structures and prompt timings affect data quality in event-based, repeated ITM surveys. Through a randomized experiment, our study seeks to clarify their impacts and provide research findings that can inform the design of improved ITM surveys.

## Methods

### Experimental design

We implemented a fully crossed 2 × 2 factorial experimental design, randomly assigning participants to one of the four conditions that varied both the incentive structure and survey timing. The incentive factor included two conditions. In the per-survey condition, participants were promised $2 for each survey that they completed. In the daily condition, participants were promised $4 for each day in which they completed at least one survey. In both conditions, incentives were provided in the form of an e-gift card (Amazon) and were capped at $56.

The survey timing factor also included two conditions. In the near-time condition, survey invitations were sent via the research app immediately after a trip had ended. In the in situ condition, survey invitations were sent during a trip, approximately three stops before each participant’s pre-declared stop, allowing adequate time to complete the survey.^2^ The app utilized geolocation data to determine when participants were near their designated stop. In both conditions, separate survey invitations were sent for each trip segment, accommodating participants who transferred between public transit lines. Survey invitations were visible for up to 30 min after being issued. All study procedures were approved by the institutional review board at the University of Maryland (approval: 2011451–5).

### Surveys

**Fig. 1:**
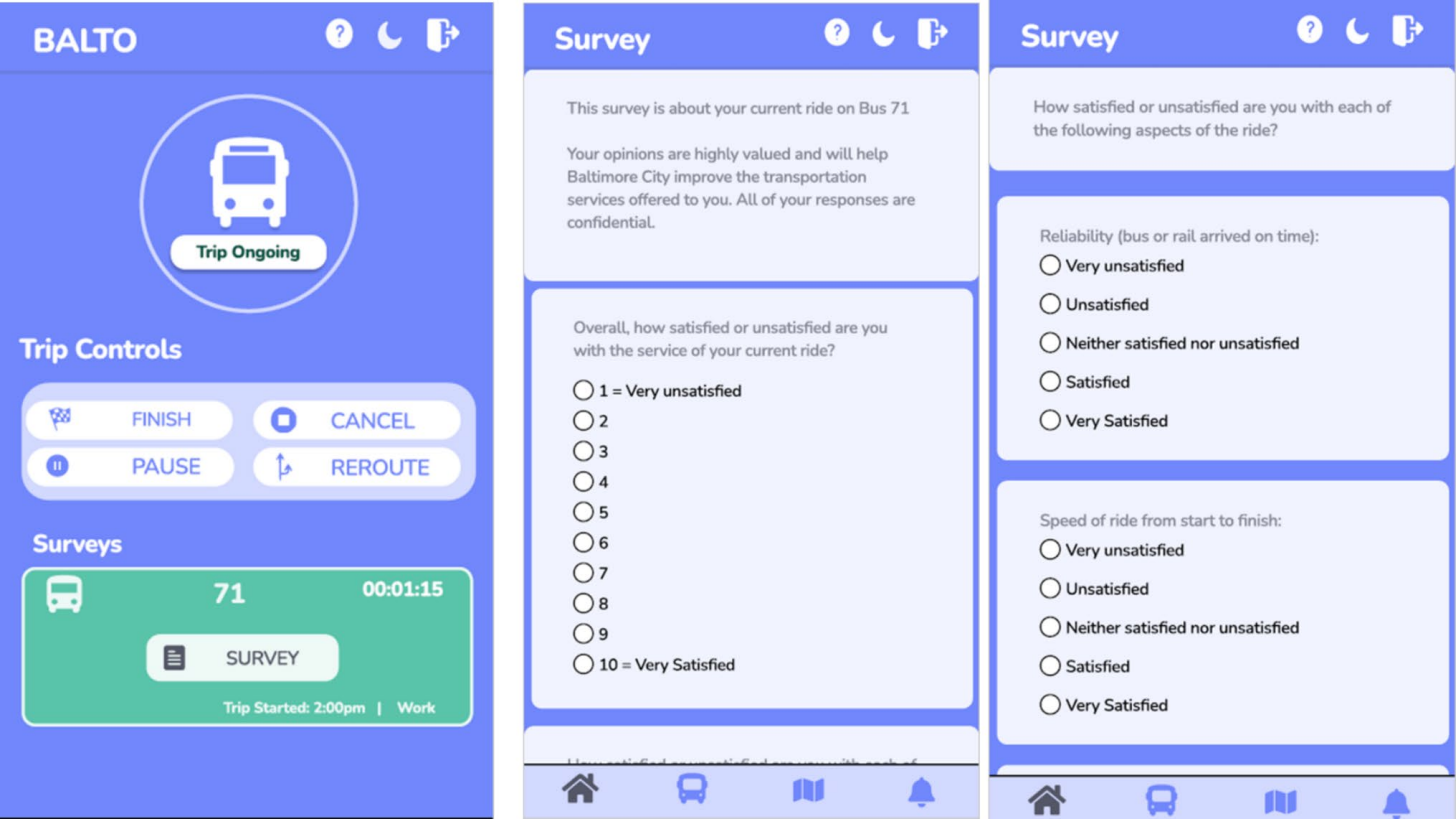
Survey prompt (left panel, near the bottom of the screen) and questionnaire (middle and right panels)

The trip assessment questionnaire contained a variety of items used in previous studies to measure transit rider satisfaction (Glascock, 1997; Stuart et al., 2000; Weinstein, 2000; Zhou et al., 2004; NASEM, 2005; Minser & Webb, 2010; Zhao et al., 2014). This included an overall satisfaction rating question on a 10-point scale (“1 = very unsatisfied”; “10 = very satisfied”); seven items about specific trip aspects (comfort, cleanliness, safety, reliability, courtesy of driver, speed, and availability of seats) rated on fully labeled five-point scales (“very unsatisfied” to “very satisfied); and an open-ended question for additional comments (“Anything else…?”). While the questionnaire was designed to measure aspects of the trip that were expected to remain stable throughout the experience, we acknowledge that some aspects might change over the course of the trip.^3^ Figure 1 presents example screenshots of the survey prompt and the questionnaire. The complete questionnaire is provided online in the supplementary material.^4^

### Smartphone app

A specialized smartphone app was developed to collect trip information and continuously monitor GPS during the trips. The app was developed for the Android platform, as available data suggest it is the predominant operating system among the target population of our study (Howarth, 2025). Due to resource and timeline constraints, cross-platform development was not feasible for this study (see Frias-Martinez et al., 2023, for details on how the app was developed).

The app collected information about the type of transit used (e.g., bus, light rail), transfers between transit lines (e.g., changing buses), and destinations. Participants downloaded the app from the Google Play store, entered a registration code, provided consent by acknowledging the consent form, submitted an email address, and answered three demographic questions (gender, age, race/ethnicity). Email addresses were verified by sending a registration code to each address and requiring participants to enter the code into the app, ensuring one registration per address.

Following registration, participants recorded their public transit trips in Baltimore City via the app over a 2-week period. This involved entering route details and pressing “start trip” to enable location data sharing. The participants were required to manually declare trips—rather than the app automatically logging them—due to privacy considerations that aimed to prevent GPS monitoring when the participants were not actively using public transit. The app collected geolocation data until participants ended their trip. After 2 weeks, the participants were invited to complete a web survey with evaluation questions that asked about their experience with the study.

### Recruits

The participants were recruited via three methods. First, mail invitations were sent to a random sample of 2,800 addresses from households in nine lower-income Baltimore City neighborhoods which had been flagged by a sample vendor as having at least one Android device in the household.^5^ These neighborhoods were specifically targeted for this project in order to study public transit use among underserved communities. Second, project staff distributed recruitment flyers at 14 tabling events near transit stations within the same neighborhoods and at community events. Finally, 126 advertisements were placed inside buses and bus shelters in and around the same neighborhoods. These ads featured a quick-response (QR) code and a shortened URL (Bitly) that linked to an enrollment webpage in Qualtrics. The webpage displayed participation instructions to the recruits after they entered their email address, and these instructions were also sent via email. An analysis of IP addresses in Qualtrics identified seven individuals who downloaded the app using different emails; only data from their initial download were retained. See Antoun et al. (2025) for more details about the recruitment methods used for this study.

Participants were randomized into experimental conditions through unique registration codes corresponding to one of the four conditions provided in the recruitment materials. The text for each condition was as follows:

- **Incentive structure**: You will earn [“$2 for each survey completed on time” OR “$4 per day with a survey completed on time”], with a maximum potential earning of $56. Surveys must be completed within 30 min after they are launched in the app to be considered on time.
- **Survey timing:** Short surveys about your trip will appear in the app [“while you are aboard” OR “at the end of the trip, after you press the FINISH button”].

Participants were recruited between September 2023 and January 2025. Each participant completed a 2-week data collection period immediately following their enrollment. In total, 610 participants downloaded the app and completed the initial registration steps. As shown in Table 1, there were no significant differences in participant characteristics (age, gender, and race/ethnicity) or recruitment sources across the experimental groups at the *p* <. 05 level.^6^

**Table 1:** Comparisons of respondent demographics and recruitment sources across the four experimental conditions

Variables	Daily incentive/ In situ prompt *n* = 147	Daily incentive/ Near-time prompt *n* = 149	Per-survey incentive/ In situ prompt *n* = 162	Per-survey incentive/ Near-time prompt *n* = 152	Statistical test and ***p***-value
Age					
Mean age	40.2	38.3	41.4	40.9	*F* = 1.75, *p* =.16
Gender (%)					
Female	60.5	55.0	60.5	53.3	χ^2^(6) = 4.42, *p* =.62
Male	34.0	41.6	36.4	42.8	
Other	5.4	3.4	3.1	3.9	
Race (%)					
Black/African American	63.3	61.1	61.1	59.2	χ^2^(6) = 11.38, *p* =.08
Other	17.0	26.2	17.9	15.8	
White/Caucasian	19.7	12.8	21.0	25.0	
Recruitment method (%)					
Advertisement	76.9	82.6	82.7	81.6	χ^2^(6) = 3.46, *p* =.75
In-person	8.2	4.0	5.6	6.6	
Mailing	15.0	13.4	11.7	11.8	

Of the 610 participants who downloaded the app, 226 recorded at least one trip, and 175 completed at least one survey. In total, participants were invited to complete 1,739 surveys, and 1,146 of those surveys were completed. Among those recording trips, 41.1% recorded 1 trip, 11.5% recorded 2, 18.1% recorded 3–5, 15.9% recorded 6–10, and 13.7% recorded 11 or more. For those who completed surveys, 34.3% completed 1, 16.0% completed 2, 14.3% completed 3–5, 16% completed 6–10, and 19.4% completed 11 or more. The number of trips and surveys completed by participants ranged from 1 to 41 and 1 to 47, respectively. These variables were included as participant-level covariates in our analysis to account for variation in trip and survey completion. Regarding survey timing, we acknowledge that some in situ participants may have completed surveys after their trip ended. However, the available evidence suggests that this was not very common. Participants had an estimated 10–15 min of remaining travel time after receiving the invitation (see footnote 2). Survey timing data show that 58.8% of in situ surveys were completed within 5 min of the invitation, 76.1% within 10 min, and 85.5% within 15 min. These patterns indicate that most respondents likely completed surveys during transit.

### Measures

To test our hypotheses, we developed six outcome measures. The following four measures were calculated for each participant.

1. *Days of participation:* The number of days each participant completed at least one survey during their 2-week study period, with values ranging from 0 to 14.
2. *Surveys completed:* The total number of surveys each participant completed during the study period.
3. *Response rate:* Calculated using a binary variable indicating whether each survey sent to participants in the app was completed.
4. *Response differentiation across trips.* The average participant-level standard deviation for each of the eight satisfaction items among those who completed two or more surveys was calculated. A score of 0 for an item indicates that a respondent gave the same rating for that item on every trip during the study period, whereas a higher score indicates variation in survey ratings across trips. While in principle, similar responses for truly similar trips (e.g., the same route taken within a short time interval) would be coherent, we interpret greater variation in responses across trips as reflecting higher-quality answers. This is because the trip characteristics we measured (e.g., speed) were expected to differ across routes and to change during the study period even for routes traveled multiple times. In addition, as described earlier in the introduction, prior research demonstrates that when respondents report on events further in the past, they tend to rely on generalized, typical experiences rather than trip-specific details, which artificially reduces response variation.

The final set of measures consists of two indicators of satisficing behavior, which are calculated for each participant for each individual survey.

5. *Speeding:* A binary indicator of whether a participant completed the survey in less time than it would have taken to read the questions at a reading speed of 200 ms per word, the typical reading speed among college students for comprehension (e.g., Carver, 1992). The word count used for this threshold purposely excluded the initial instructions, as respondents completing the same survey multiple times might skip rereading them. We acknowledge, however, that even with this conservative threshold intended to flag only those respondents who were clearly not reading the questions, using a fixed threshold does not account for legitimate changes in completion time over repeated administrations of the survey as participants become more familiar with it, skip rereading other parts of it, or complete it in different contexts.
6. *Straightlining:* A binary indicator of whether a participant selected the same response option for all seven five-point satisfaction items in the survey.

In addition, we examined participants’ subjective experience with an evaluation question about their overall ease of completing surveys for each trip (very difficult; somewhat difficult; neither easy nor difficult; somewhat easy; very easy). Because there were relatively few negative evaluations, we recoded the variable by combining the two “difficult” responses (very difficult and somewhat difficult) into a single category to create a more balanced distribution.

### Analysis

For outcome measures at the participant level, we conducted one-way analysis of variance (ANOVA) or chi-square tests to assess significant differences in our outcomes across experimental conditions. To estimate the average treatment effect of one experimental factor while controlling for the other, we applied Poisson and linear regression models. Model fit was evaluated using the Akaike information criterion (AIC) and Bayesian information criterion (BIC), and the model with the lowest AIC and BIC values was selected for the final analysis.

For outcomes at the survey level, we used a hierarchical dataset in which surveys were nested within participants. To address the fact that participants could complete multiple surveys, we fitted marginal logistic regression models with generalized estimating equations (GEE), using the geepack package in R (Højsgaard et al., 2006). The GEE produces population-averaged estimates and robust standard errors when analyzing correlated data—such as repeated responses from the same participant—by explicitly modeling the correlation structure among observations. To identify the best correlation structure for repeated surveys within participants, we compared three working correlation structures: exchangeable, independent, and first-order autoregressive [AR(1)]. These structures were evaluated using the quasi-likelihood under the independence model criterion (QIC), and the one that provided the best fit (lowest QIC) was used in the final analysis. In addition, we examined potential interactions between incentive structure and survey timing in all these analyses, as their interplay might influence our outcome measures.

We assessed response differentiation across trips using two complementary approaches. First, we compared the average participant-level standard deviations for each of the eight public transit satisfaction items across survey timing conditions, using one-way ANOVA and pairwise *t*-tests. Second, we fitted linear mixed models for each satisfaction item, specifying fixed effects for the experimental factors and random intercepts for both trips and participants. For each outcome, we estimated two models: one assuming equal (homogeneous) error variance and another allowing error variance to differ (heterogeneous) across survey timing conditions. In this context, a larger error variance in one timing condition relative to the other indicates greater response differentiation across trips in that condition; accordingly, we anticipated larger error variance in the in situ condition than in the near-time condition. Model pairs were compared using AIC, BIC, and log likelihood, and likelihood ratio tests were performed on log likelihood differences to assess whether allowing for heterogeneous error variance significantly improved model fit. Significant differences in model fit indicate differences in response differentiation across survey timing conditions. We further examined these differences by estimating the error variances for each timing condition and testing for significant differences using *F* tests.

All analyses were conducted using R version 4.4.2. The scripts and data are available in the supplementary material online.

## Results

### Hypothesis 1: Incentives and participation days

In H1, we hypothesized that daily incentives would increase the number of days participants completed at least one survey compared with per-survey incentives. The results supported this hypothesis, showing that participants in the daily incentive condition completed surveys on significantly more days. On average, participants completed surveys on 0.78 days when offered per-survey incentives compared with 1.13 days when offered daily incentives, *F*(1, 608) = 3.91, *p* =.048. As shown in Table 2, this pattern holds in a Poisson regression model, which estimates the average treatment effect of the incentive structure while controlling for survey timing. The model indicates that participants offered the daily incentive completed surveys on 45% more days than those offered the per-survey incentive.

**Table 2:** Poisson regression model of participant survey completion days by incentive structure and survey timing

	Incidence rate ratio	95% CI	***p***
Intercept	0.73	0.62, 0.85	** <.01**
Incentive structure: Daily	1.45	1.23, 1.72	** <.01**
Survey timing: In situ	1.14	0.97, 1.35	.11

*N* = 610 participants

### Hypothesis 2: Incentives and surveys completed

We expected that per-survey incentives would lead to a higher total number of surveys completed by participants. However, our analyses did not support this hypothesis. On average, participants in the per-survey condition completed 1.56 surveys, while those in the daily incentive condition completed 2.22 surveys. This difference was not statistically significant, *F*(1, 608) = 2.31, *p* =.13.^7^ The linear regression model results are consistent with this conclusion (Appendix Table 6), indicating that the incentive structure had no significant effect on the total number of surveys completed.

### Hypothesis 3: Survey timing and response rates

H3 proposed that in situ prompts would lead to higher survey response rates than near-time prompts. As expected, survey response rates were higher in the in situ condition than in the near-time condition (74.7% vs. 58.0%), χ^2^(1) = 53.38, *p* <.01, a difference of 16.7 percentage points. As shown in Table 3, this pattern is supported by a marginal logistic regression model, which estimates the population-average effect of prompt timing on survey completion. The model indicates that participants in the in situ condition had 2.46 times greater odds of completing each survey they were invited to than those in the near-time condition.

**Table 3:** Marginal logistic regression of the effects of incentive structure and survey timing on survey completion odds

	Odds ratio	95% CI	***p***
Intercept	0.47	0.27, 0.83	** <.01**
Incentive structure: Daily	1.06	0.65, 1.72	.83
Survey timing: In situ	2.46	1.47, 4.14	** <.01**
Number of trips taken	1.10	1.06, 1.14	** <.01**

*n* = 1,729 triggered surveys, *N* = 226 participants. We used an independent correlation structure

We also explored whether the effect of survey timing on response rates was moderated by time of day, based on the reasoning that in situ surveys might be more difficult to complete during peak transit hours. To test this, we created a binary variable indicating whether the trip was recorded during rush hour (defined as 7:00–10:00 AM and 3:30–7:00 PM) and tested the interaction between rush hour status of trips and survey timing. The results showed no significant interaction between survey timing and rush hour status (odds ratio [OR] = 1.45, *p* =.26; Appendix Table 7), indicating that the higher response rates for in situ prompts held consistently regardless of whether the trip took place during peak or off-peak hours.

### Hypothesis 4: Survey timing and satisficing

This hypothesis examines whether respondents engage in fewer satisficing behaviors when they answer questions during their trips than they do after their trips. Consistent with our expectations, participants were less likely to speed through the surveys in the in situ condition (39.8%) than in the near-time condition (47.3%), χ^2^(1) = 6.25, *p* =.01. Because participants tended to complete surveys more quickly as they gained experience, we controlled for the total number of surveys completed by each participant using a marginal model. The results indicate that the lower likelihood of speeding in the in situ condition persisted after accounting for this factor, with in situ prompts associated with marginally lower odds of speeding (OR = 0.65, *p* =.07; Appendix Table 8). Descriptive analysis also revealed differences in straightlining (providing the same response across items), with in situ surveys showing a rate of 38.8% compared to 28.3% in the near-time condition, χ^2^(1) = 13.4, *p* <.01. However, this difference was no longer significant after controlling for the number of surveys completed (OR = 1.12, *p* =.66; Appendix Table 9).

We also investigated whether commuting congestion moderated the effect of survey timing on satisficing behaviors by testing the interaction between rush hour status and survey timing for both satisficing indicators. No significant interactions were observed in either model (Tables S1 and S2 in the online supplementary material), indicating that the reduction in speeding associated with in situ prompts was consistent across both rush hour and non-rush hour trips.

### Hypothesis 5: Survey timing and response differentiation across trips

Our final hypothesis posited that in situ prompts would lead to greater response differentiation across trips than would near-time prompts. To test this, we first calculated the average participant-level standard deviation for each of the eight satisfaction items among participants who completed two or more surveys and compared these means across survey timing conditions using *t*-tests. Six out of eight estimates were in the expected direction, with in situ surveys yielding more varied responses than near-time surveys, although none of these differences reached statistical significance (Appendix Table 10).

Next, we fit linear mixed models for each satisfaction item, specifying fixed effects for survey timing and incentive structure and random intercepts for trips and participants. We compared models assuming homogeneous error variance with those allowing for heterogeneous error variance across survey timing conditions. The likelihood ratio tests indicated significant improvements in model fit for seven of the eight satisfaction items when heterogeneous error variance was allowed, as shown in Table 4. This indicates differing levels of response differentiation across trips between survey timing conditions.

**Table 4:** Likelihood ratio tests and model fit indices for linear mixed models: homogeneous vs. heterogeneous error variance in satisfaction items

	Homogenous error			Heterogeneous error			L Ratio	***p***
Survey item	AIC	BIC	logLik	AIC	BIC	logLik		
Overall satisfaction	4876	4906	− 2432	4870	4905	− 2428	7.32	** <.01**
Reliability	3349	3379	− 1668	3346	3381	− 1666	4.48	**.03**
Speed	2981	3011	− 1484	2979	3014	− 1482	4.14	**.04**
Safety	2698	2728	− 1343	2689	2724	− 1337	10.68	** <.01**
Courtesy	2881	2911	− 1435	2863	2898	− 1425	19.98	** <.01**
Cleanliness	3000	3030	− 1494	3000	3035	− 1493	1.52	.22
Comfort	2821	2851	− 1404	2814	2849	− 1400	8.47	** <.01**
Available seats	3108	3138	− 1548	3092	3127	− 1539	18.30	** <.01**

AIC: Akaike information criterion; BIC: Bayesian information criterion; logLik: log-likelihood; L Ratio: likelihood ratio test. Lower AIC and BIC values and higher logLik values indicate better model fit. *N* = 175 participants; *n* = 1,146 completed surveys for all the models

Finally, using the heterogeneous models, we estimated the error variance for each timing condition and conducted *F*-tests to compare them. As shown in Table 5, all eight estimates were in the expected direction, with in situ surveys exhibiting more participant-level response variation than near-time surveys. Moreover, all the *F*-tests were statistically significant, providing consistent support for Hypothesis 5 that in situ surveys would lead to more differentiated responses across trips.

**Table 5:** *F-*tests comparing the error variance of satisfaction items from heterogeneous linear mixed models across survey timing conditions

Survey item	Near-time error SD	In situ error SD	*F*(640,504), *p*
Overall satisfaction	1.41	1.66	1.39, **<.01**
Reliability	0.80	0.90	1.28, **<.01**
Speed	0.68	0.76	1.25, ** <.01**
Safety	0.51	0.62	1.51, ** <.01**
Courtesy	0.57	0.74	1.69, **<.01**
Cleanliness	0.65	0.70	1.17, **.03**
Comfort	0.64	0.74	1.35, **<.01**
Available seats	0.70	0.88	1.56, ** <.01**

#### Additional analyses

Lastly, we tested whether the effect of survey timing on our data quality indicators depended on the incentive structure, or vice versa. Across our analyses, only two interactions were statistically significant. The effect of the daily incentive (compared with the per-survey incentive) on participation days was smaller for participants who answered in situ. Additionally, an interaction for speeding was found, indicating that the effect of in situ prompts (compared with near-time prompts) was smaller for those receiving per-survey incentives. Overall, however, the relationships between the data quality measures and each experimental factor were largely consistent across the levels of the other factor.

Finally, we evaluated whether survey timing influenced participants' perceptions of the ease or difficulty of participating in the study. Compared with participants in the near-time condition, those in the in situ condition were slightly more likely to rate their experience as “very easy” (21.4% vs. 20.2%) and less likely to rate it as “difficult” (7.1% vs. 11.3%). However, these differences were not statistically significant (χ^2^ = 3.38, *df* = 3, *p* = 0.34).

## Discussion

ITM surveys provide valuable opportunities for collecting data in real time, but they also present significant design challenges. Our experimental study produced several findings on how incentive structures and survey timing can impact data quality in these surveys. As predicted by Hypothesis 1, daily incentives significantly increased the number of days participants completed at least one survey compared with per-survey incentives. Consistent with Hypothesis 3, issuing in situ prompts led to higher survey completion rates than near-time prompts. We found partial support for Hypothesis 4, as in situ prompts reduced speeding when participants answered the survey questions. Finally, supporting Hypothesis 5, in situ prompts resulted in more differentiated responses across trips, suggesting improved accuracy in capturing event-specific details compared with near-time reports.

The effectiveness of the daily incentive likely stems from offering a larger reward for the first survey of each day ($4 vs. $2). Contrary to expectations, this incentive did not result in a significant decrease in the total number of surveys completed. This may be because participants typically completed only one or fewer surveys per day, so the daily incentive did not limit additional rewards as it might in studies where participants complete multiple surveys each day. It is worth noting that overall participation, measured by the number of days participants completed at least one survey, was low in both incentive conditions. This likely indicates that participants either did not ride public transit frequently or did not report every trip. These findings suggest that, in hindsight, our goal of encouraging daily participation was not well aligned with the actual transit habits. Future efforts should incorporate realistic estimates of event frequency (e.g., using reliable auxiliary information) when developing participation goals and corresponding incentive structures. 

The higher survey completion rates with in situ prompts, even during rush hour, can be attributed to the interruptible nature of transit rides compared with post-trip activities. This finding supports the general notion that respondents are willing to complete in situ app-based surveys during activities requiring minimal cognitive effort, particularly when they are already using their phones (Ochoa & Revilla, 2023; Yan et al., 2021). Furthermore, participants may have found the survey topic—their transit experience—more interesting while still involved in the activity.

The observed reduction in speeding when participants answered questions during their trips, as opposed to afterward, aligns with the idea that responding in situ is cognitively easier than recalling information retrospectively. Consequently, respondents may have been less likely to rely on cognitive shortcuts (such as speeding) when completing surveys during the trip. Although we did not observe consistent differences in straightlining by timing condition, this may indicate that respondents did not find the questions particularly difficult, even when answering them after a trip. This could be attributed to the relatively small number of questions and the short recall period (i.e., within 30 min of trip completion).

The greater response differentiation observed for in situ surveys—perhaps the study’s most important finding—demonstrates their capacity to capture distinct trip experiences. This outcome is consistent with theories of the cognitive response process, which suggest that reporting on one’s immediate surroundings enhances response accuracy and that even brief delays can cause memory degradation for routine events (Means & Loftus, 1991; Schwarz, 2012). Although the benefits of real-time reporting are often noted, there is limited empirical research directly comparing true in situ reports to any type of retrospective reporting, whether near or far removed from the event. Recent studies that have begun to make these comparisons have found some positive effects of in situ reporting on response quality, including more detailed open-ended responses and more accurate factual reports of event details, such as the temperature during a beach visit (Ochoa, 2025a, b). Our study’s finding of increased differentiation for in situ reports further underscores their effectiveness for capturing context-specific details and reducing recall bias.

Our findings offer two practical recommendations for researchers designing ITM surveys that are repeated over time. First, there is a clear benefit to using daily incentives to encourage consistent participation, particularly when respondents complete a relatively small number of surveys each day. Second, administering surveys in situ can improve data quality in some circumstances. These likely include contexts where respondents are engaged in activities that are easily interruptible, where questions relate directly to the immediate context, and where specific details are prone to being quickly forgotten.

This study has several limitations to consider. By offering only an Android app, we restricted our recruitment to individuals with access to an Android device, who make up slightly less than half of US smartphone users and generally have lower incomes than iPhone users (Howarth, 2025). A web-based option that does not require downloading an app may increase participation in future studies. Travel experiences represent just one of many possible constructs to measure with ITM surveys, and other events, behaviors, and experiences might yield different results. In addition, our focus on Baltimore City may limit the generalizability of our findings to other locations. For example, in cities with more crowded buses and trains, individuals might engage with their phones less frequently while on board, potentially leading to different outcomes. The participants also completed a relatively small average number of surveys, primarily early in the study period, which limited our statistical power to detect overall differences and constrained our ability to evaluate whether the magnitude or nature of the experimental effects varied over the course of the study. Requiring respondents to self-declare trips, due to privacy considerations, meant that unrecorded trips did not trigger survey invitations and could not be included in response rate calculations. Future studies could explore ways of using geolocation data for passive event detection while protecting participant privacy (e.g., automatically deleting GPS data not associated with public transit trips). Finally, our assessment of response quality was based on indicators of measurement error rather than direct measurements of reliability and validity. Additionally, it did not address some complexities of repeated measurements, such as potential reactivity or the possibility that in situ reports may miss details occurring after reporting but before the event concludes (see, e.g., König et al., 2022).

We encourage further research to address these limitations, including conducting replications in different settings and exploring a wider range of activities that trigger surveys. Future studies could extend the analysis of contextual factors beyond peak versus nonpeak travel hours, which did not moderate effects in our study, possibly because it was only a weak proxy for the actual crowdedness of buses and trains. Researchers might consider factors that make activities harder to interrupt or the survey-taking environment more distracting, such as the presence of young children or other people with the respondent. Assessing the impacts of these additional variables could provide deeper insights into participants’ likelihood of completing ITM surveys across different contexts and under what conditions this approach improves response accuracy.

In conclusion, we hope our findings—demonstrating higher response rates and improved response quality for in situ surveys in certain contexts—will encourage researchers to consider this technique for real-time assessment of opinions and behaviors. Particularly given the widespread use of smartphones, which enable the deployment of specialized research apps, social researchers stand to benefit from the increased use of ITM surveys. Broadening the adoption of these methods has the potential to deepen our understanding of respondents’ experiences in the context of specific, real-world events.

## Data Availability

The data and materials for the experiment are available as supplemental material here: https://osf.io/98qbp/.

## Appendix

**Table 6:** Linear regression of survey timing and incentive structure on the number of surveys completed by participants

	Coefficient	95% CI	***p***
Intercept	1.35	0.61, 2.09	** < 0.01**
Incentive structure: Daily	0.67	− 0.18, 1.52	.12
Survey timing: In situ	0.41	− 0.44, 1.26	.35

*N* = 610 participants

**Table 7:** Marginal logistic regression of survey timing and rush hour interaction on survey completion odds

	Odds ratio	95% CI	***p***
Intercept	0.45	0.27, 0.75	** <.01**
Survey timing: In situ	2.04	1.14, 3.65	**.02**
Trip during rush hour: Yes	1.19	0.75, 1.90	.46
Number of trips taken	1.10	1.06, 1.14	** <.01**
Survey timing × Trip during rush hour: (In situ/Yes)	1.45	0.73, 2.91	.29

*N* = 226 participants,* n* = 1,729 triggered surveys. We used an independent correlation structure

**Table 8:** Marginal logit model predicting speeding odds by incentive structure and survey timing, controlling for the number of surveys completed

	Odds ratio	95% CI	***p***
Intercept	0.28	0.17, 0.47	** <.01**
Incentive structure: Daily	1.39	0.87, 2.23	.17
Survey timing: In situ	0.65	0.40, 1.04	.07
Number of surveys completed	1.06	1.03, 1.09	** <.01**

*N* = 175 participants,* n* = 1,146 completed surveys. We used an exchangeable correlation structure

**Table 9:** Marginal logit model predicting straightlining odds by incentive structure and survey timing, controlling for the number of surveys completed

	Odds ratio	95% CI	***p***
Intercept	0.22	0.13, 0.39	** <.01**
Incentive structure: Daily	1.00	0.61, 1.63	.99
Survey timing: In situ	1.12	0.67, 1.87	.66
Number of surveys completed	1.04	1.02, 1.07	** <.01**

*N* = 175 participants,* n* = 1,146 completed surveys. We used an exchangeable correlation structure

**Table 10:** Average participant-level standard deviations of satisfaction ratings across survey timing conditions

Outcome variable	Average SD Near-time	Average SD In situ	*t*-statistic	*p*
Overall satisfaction	1.47	1.58	− 0.43	.67
Reliability	0.83	0.79	0.35	.73
Speed	0.61	0.70	− 0.86	.39
Safety	0.48	0.66	− 1.79	.08
Courtesy	0.57	0.73	− 1.55	.12
Cleanliness	0.65	0.64	0.10	.92
Comfort	0.56	0.64	− 0.93	.36
Available seats	0.72	0.88	− 1.37	.17

*N* = 115 participants who completed two or more surveys; *n* = 1,086 completed surveys

## Footnotes

1 Although “near-time” is not a standard term in this context, we use it to indicate that the prompt was sent immediately after an event, rather than during the event or after a delay.

2 We estimated that a bus traveling three stops—covering an average distance of 3.3 miles—with two intermediate stops each lasting 1 min, would require approximately 10–15 min to complete the trip. This estimate assumes a travel speed of 15–25 mph (depending on congestion levels), which reflects real-world data (TomTom, 2025).

3 We also acknowledge an oversight in the wording of the item concerning “speed of ride,” which refers to the trip from “start to finish.” This may not be appropriate if the trip has not yet concluded when the survey is completed.

4 https://osf.io/98qbp/

5 The vendor started with the US Postal Service's Computerized Delivery Sequence File (CDSF) for the target neighborhoods and appended up to three cell phone numbers along with the type of smartphone associated with each number when possible. Approximately half of addresses with matched numbers were associated with at least one Android device; these household constituted the sample frame.

6 Our sample size (about 152 per condition) provides 80% power at a 5% significance level to detect a difference of approximately 0.32 standard deviations between any two conditions; for a binary outcome with a baseline of 50%, this corresponds to an approximately 15-percentage-point difference. For a test of a main effect (collapsing across the other factor), our sample size (about 304 per group) provides 80% power to detect a difference of approximately 0.23 standard deviations; for a binary outcome, this corresponds to detecting a difference as small as approximately 10–11 percentage points.

7 A supplementary analysis examined the total number of trips declared by each participant during the study period—including trips for which no surveys were completed. Results mirrored the main findings: participants in the per-survey incentive condition reported an average of 1.57 trips, while those in the daily incentive condition reported 2.19 trips, *t*(608) =  − 1.68, *p* =.09.

## Code availability

The R code used for analysis is available in the online supplementary material at https://osf.io/98qbp/.

## Declarations

### Open practices statement

This study design and analysis was not preregistered.
